# Oleic acid is an endogenous ligand of TLX/NR2E1 that triggers hippocampal neurogenesis

**DOI:** 10.1073/pnas.2023784119

**Published:** 2022-03-25

**Authors:** Prasanna Kandel, Fatih Semerci, Rachana Mishra, William Choi, Aleksandar Bajic, Dodge Baluya, LiHua Ma, Kevin Chen, Austin C. Cao, Tipwarin Phongmekhin, Nick Matinyan, Alba Jiménez-Panizo, Srinivas Chamakuri, Idris O. Raji, Lyra Chang, Pablo Fuentes-Prior, Kevin R. MacKenzie, Caroline L. Benn, Eva Estébanez-Perpiñá, Koen Venken, David D. Moore, Damian W. Young, Mirjana Maletic-Savatic

**Affiliations:** ^a^Integrative Molecular and Biomedical Sciences Graduate Program, Baylor College of Medicine, Houston, TX 77030;; ^b^Department of Pharmacology & Chemical Biology, Baylor College of Medicine, Houston, TX 77030;; ^c^Department of Pediatrics–Neurology, Baylor College of Medicine, Houston, TX 77030;; ^d^Jan and Dan Duncan Neurological Research Institute, Texas Children’s Hospital, Houston, TX 77030;; ^e^Medical Scientist Training Program, Baylor College of Medicine, Houston, TX 77030;; ^f^Department of Molecular and Human Genetics, Baylor College of Medicine, Houston, TX 77030;; ^g^Department of Interventional Radiology, University of Texas MD Anderson Cancer Center, Houston, TX 77030;; ^h^NMR and Drug Metabolism Advanced Technology Cores, Baylor College of Medicine, Houston, TX 77030;; ^i^Department of Biosciences, Rice University, Houston, TX 77005;; ^j^Department of Chemistry, Rice University, Houston, TX 77005;; ^k^Verna and Marrs McLean Department of Biochemistry and Molecular Biology, Baylor College of Medicine, Houston, TX 77030;; ^l^Department of Biochemistry and Molecular Biomedicine, Institute of Biomedicine, University of Barcelona, 08028 Barcelona, Spain;; ^m^Department of Pathology and Immunology, Baylor College of Medicine, Houston, TX 77030;; ^n^Department of Molecular and Cellular Biology, Baylor College of Medicine, Houston, TX 77030;; ^o^Molecular Basis of Disease, Biomedical Research Institute Sant Pau, 08025 Barcelona, Spain;; ^p^Pfizer Regenerative Medicine, Cambridge CB21 6GP, United Kingdom;; ^q^Center for Drug Discovery, Baylor College of Medicine, Houston, TX 77030;; ^r^Department of Neuroscience, Baylor College of Medicine, Houston, TX 77030

**Keywords:** TLX, NR2E1, fatty acids, neural stem/progenitor cells, neurogenesis

## Abstract

Neural stem cells, the source of newborn neurons in the adult hippocampus, are intimately involved in learning and memory, mood, and stress response. Despite considerable progress in understanding the biology of neural stem cells and neurogenesis, regulating the neural stem cell population precisely has remained elusive because we have lacked the specific targets to stimulate their proliferation and neurogenesis. The orphan nuclear receptor TLX/NR2E1 governs neural stem and progenitor cell self-renewal and proliferation, but the precise mechanism by which it accomplishes this is not well understood because its endogenous ligand is not known. Here, we identify oleic acid (18:1ω9 monounsaturated fatty acid) as such a ligand. We first show that oleic acid is critical for neural stem cell survival. Next, we demonstrate that it binds to TLX to convert it from a transcriptional repressor to a transcriptional activator of cell-cycle and neurogenesis genes, which in turn increases neural stem cell mitotic activity and drives hippocampal neurogenesis in mice. Interestingly, oleic acid-activated TLX strongly up-regulates cell cycle genes while only modestly up-regulating neurogenic genes. We propose a model in which sufficient quantities of this endogenous ligand must bind to TLX to trigger the switch to proliferation and drive the progeny toward neuronal lineage. Oleic acid thus serves as a metabolic regulator of TLX activity that can be used to selectively target neural stem cells, paving the way for future therapeutic manipulations to counteract pathogenic impairments of neurogenesis.

## Significance

Adult hippocampal neurogenesis underpins learning, memory, and mood but diminishes with age and certain illnesses. The orphan nuclear receptor TLX/NR2E1 regulates neural stem and progenitor cell self-renewal and proliferation, but its orphan status has hindered its utilization as a therapeutic target to modulate adult neurogenesis. Here, we deorphanize TLX and report that oleic acid is an endogenous, metabolic ligand of TLX. These findings open avenues for future therapeutic modulation of TLX to counteract cognitive and mental decline in aging and diseases associated with decreased neurogenesis.

The mammalian brain contains a population of neural stem cells within the hippocampus that produce functional neurons well into adulthood (1–4). This ongoing capacity to form new neurons, known as adult neurogenesis, enables learning and memory (5, 6) and supports proper mood regulation (7). Conversely, impairments in neural stem cell proliferative capacity and neuronal commitment are associated with advanced age (8, 9) and disorders such as depression (10) and Alzheimer’s disease (11, 12). Indeed, antidepressant therapies such as serotonin reuptake inhibitors are effective only insofar as they succeed in stimulating neurogenesis (7). There has thus been considerable interest in learning how to manipulate hippocampal neurogenesis to treat a range of conditions.

One key question is how to preserve the pool of neural stem and progenitor cells while promoting the ongoing generation of neurogenic progeny. To maintain their population, neural stem and progenitor cells must be able to self-renew, and it is well established that their self-renewal and proliferation are regulated by the nuclear receptor TLX (also known as NR2E1) (13–15). Nuclear receptors are transcription factors whose functions are modulated through the binding of endogenous small molecules. TLX is considered an orphan nuclear receptor since its endogenous ligand has not yet been discovered [exogenous and synthetic ligands have been reported but without physiological function in vivo (16–19)]. The identification of such a functional endogenous ligand would help validate TLX as a therapeutic target for promoting neurogenesis for a variety of human diseases (10–12) and potentially also suggest approaches to modulate ligand abundance or availability.

Several clues have guided our studies to identify a functional biological ligand for TLX. First, nuclear receptors as a class tend to bind lipophilic molecules, such as hormones, vitamins, and fatty acids. Second, neural stem cells—like any other proliferating cells—require de novo lipogenesis to proliferate (20–25), which makes fatty acids particularly abundant in these cells. In fact, compared to other cell types in the brain such as astrocytes and fully differentiated neurons, neural stem cells have particularly high levels of monounsaturated fatty acids (MUFAs) (26). Third, we previously identified an NMR spectroscopy signal that correlates with neurogenic activity in mice and humans; it resonates at 1.28 ppm, a frequency that corresponds to the saturated hydrocarbon groups that characterize all fatty acids (26). Fourth, blocking the activity of fatty acid synthase, which catalyzes the synthesis of saturated fatty acids, markedly reduces the 1.28-ppm NMR signal (26) and prevents running from stimulating neurogenesis in mice (20). Based on these observations, we hypothesized that fatty acids could be critical to TLX function.

Interestingly, while many metabolic genes that are up-regulated in quiescent neural stem cells are associated with fatty acid synthesis, these cells have particularly high expression of stearoyl-CoA (coenzyme A) desaturases (27, 28) involved specifically in MUFA synthesis. This suggests that MUFAs might have a special role in controlling the cell cycle in neural stem cells. In this study, we asked whether MUFA synthesis is necessary for survival and proliferation of these cells, and whether these fatty acids might act as ligands for TLX in neural stem cells.

## Results

### MUFAs Are Essential for Neural Stem and Progenitor Cell Survival and Proliferation.

**Fig. 1.**
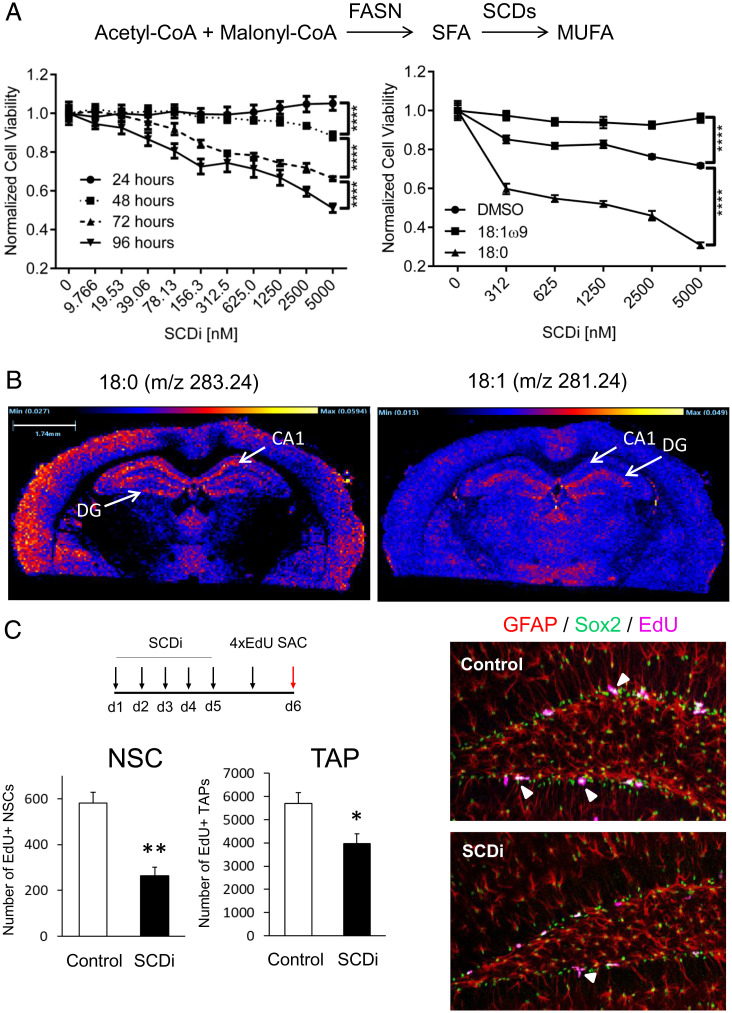
Neural stem and progenitor cells depend on de novo MUFA synthesis to survive and proliferate. (*A*) Acetyl-CoA and methyl-malonyl CoA serve as substrates for saturated fatty acid (SFA) synthesis catalyzed by fatty acid synthase (FASN). SFAs are converted to MUFAs via desaturases, such as the stearoyl-CoA desaturases (SCDs), which catalyze the rate-limiting step in MUFA synthesis. (*Left*) Dose-dependent human neural stem and progenitor cell viability following SCD inhibition with CAY10566 (SCDi), normalized to vehicle (dimethyl sulfoxide, DMSO) response. (*Right*) Dose-dependent human neural stem and progenitor cell viability following 144-h exposure to SCDi and then treated with 18:1ω9 MUFA or its precursor 18:0 SFA, normalized to the respective treatment (*n* ≥ 3 per group). Statistics was done using two-way ANOVA and Tukey's multiple comparisons test, ****P ≤ 0.0001. (*B*) Imaging mass spectrometry of the 3-mo-old wild-type C57BL/6J mouse brain shows distribution of the 18:0 SFAs (*m/z* 283.24, *Left*), the precursor of 18:1 MUFAs (*m/z* 281.24, *Right*). Dentate gyrus (DG) and CA1 region of the hippocampus are indicated (arrows). (*C*) SCDi (3 mg/kg body weight) or sham (0.03 N HCl) was delivered orally to 2- to 3-mo-old wild-type C57BL/6J mice once daily for 5 d followed by EdU (50 mg/kg body weight, four intraperitoneal injections 2 h apart on the fifth day) to label proliferating neural stem and progenitor cells in vivo (*n* = 4 per group). Mice were killed (SAC) 24 h following the first EdU injection. Total number of EdU+ neural stem cells (NSCs: GFAP+, Sox2+, EdU+) and transient amplifying neuroprogenitors (TAPs: GFAP-, Sox2+, EdU+) cells were quantified in the dentate gyri bilaterally using quantitative stereology. Bar graphs represent mean ± SEM; *P ≤ 0.05, **P ≤ 0.01. Representative confocal micrographs show proliferating neural stem and progenitor cells (arrowheads). See also *SI Appendix*, Figs. S1–S3.

While MUFAs are abundant in mouse neural stem and progenitor cells (26), we do not know whether human stem and progenitor cells have similar fatty acid content. To examine this, we performed NMR spectroscopy on purified human neuroprogenitors derived from human embryonic stem cells (*SI Appendix*). Two-dimensional ^1^H–^1^H total correlation spectroscopy (TOCSY), which identifies coupled hydrogen (^1^H) spins, revealed the monounsaturated bond of MUFAs in these cells (*SI Appendix*, Fig. S1*A*). Next, we found several saturated fatty acid precursors and MUFAs in human neuroprogenitors using gas chromatography–mass spectrometry, with oleic acid (18:1ω9) being the most abundant MUFA in these cells (*SI Appendix*, Fig. S1*B*). Like their murine counterparts (26), human neuroprogenitors require fatty acid synthase to survive: Cerulenin, an inhibitor of fatty acid synthase, decreased their viability in a dose-dependent manner (*SI Appendix*, Fig. S2*A*). Treating the cells with CAY10566 (SCDi), an inhibitor of the stearoyl-CoA desaturases that catalyze the conversion of saturated fatty acids into MUFAs, resulted in a dose- and time-dependent decrease in survival in vitro (Fig. 1*A*, *Left*). Strikingly, this impairment could be rescued by exogenous 18:1 MUFA but not 18:0 saturated fatty acids (Fig. 1*A*, *Right* and *SI Appendix*, Fig. S2*B*), suggesting that 18:1 MUFA are critical for the human neuroprogenitor survival.

If MUFA synthesis is required for survival of these cells, then MUFAs should be abundant in the dentate gyrus of the hippocampus, where neurogenesis occurs. Using imaging mass spectrometry to map lipids in the mouse brain, we detected both 18:0 saturated fatty acids (*m*/*z* = 283.24) and 18:1 MUFA (*m*/*z* = 281.24) in the dentate gyrus, the latter producing the most abundant signal (Fig. 1*B* and *SI Appendix*, Fig. S3). To estimate more precisely the 18:1 MUFA concentration in the dentate, we spiked 0.5 mM 18:1 control onto the thalamic region devoid of the signal (*SI Appendix*, Fig. S3*B*). The 18:1 MUFA ion in the dentate gyrus had relative signal intensity similar to the spiked control, indicating high concentration of this fatty acid in the dentate gyrus in vivo (*SI Appendix*, Fig. S3*B*). In addition, oral administration of CAY10566 in mice significantly reduced dentate neural stem and progenitor cell proliferation in vivo (*P* = 0.0019 and 0.0357, respectively; Fig. 1*C*), consistent with our observations in vitro. In sum, human and mouse neural stem and progenitor cells are abundant in 18:1 MUFA and require de novo MUFA synthesis for both survival and proliferation. We therefore considered the possibility that the MUFAs are not only being used for fundamental processes such as energy metabolism and lipid membrane formation but they could also serve as signaling molecules in these cells to regulate neurogenesis.

### Oleic Acid Binds to the TLX Ligand-Binding Domain.

Given that neural stem and progenitor cells required de novo MUFAs for survival and proliferation, we next investigated their ability to directly bind TLX. Because there is no crystal structure of TLX bound to ligands, we searched for a nuclear receptor with a known structure that binds fatty acids. Initial structural studies revealed HNF4α bound to fatty acids (29, 30) and functional studies indicated that HNF4α is selectively activated by exogenous linoleic acid (18:2ω6) in mammalian cells and in the liver of fed mice (31). We therefore built a homology model of the human TLX ligand-binding domain and performed molecular docking based on the X-ray crystal structure of fatty acid-bound HNF4α ligand-binding domain (Protein Data Bank ID code 1M7W) (*SI Appendix*, Fig. S4). These docking studies suggested that fatty acids could indeed fit into the binding pocket and potentially function as cognate ligands of TLX (*SI Appendix*, Fig. S4*E*).

**Fig. 2.**
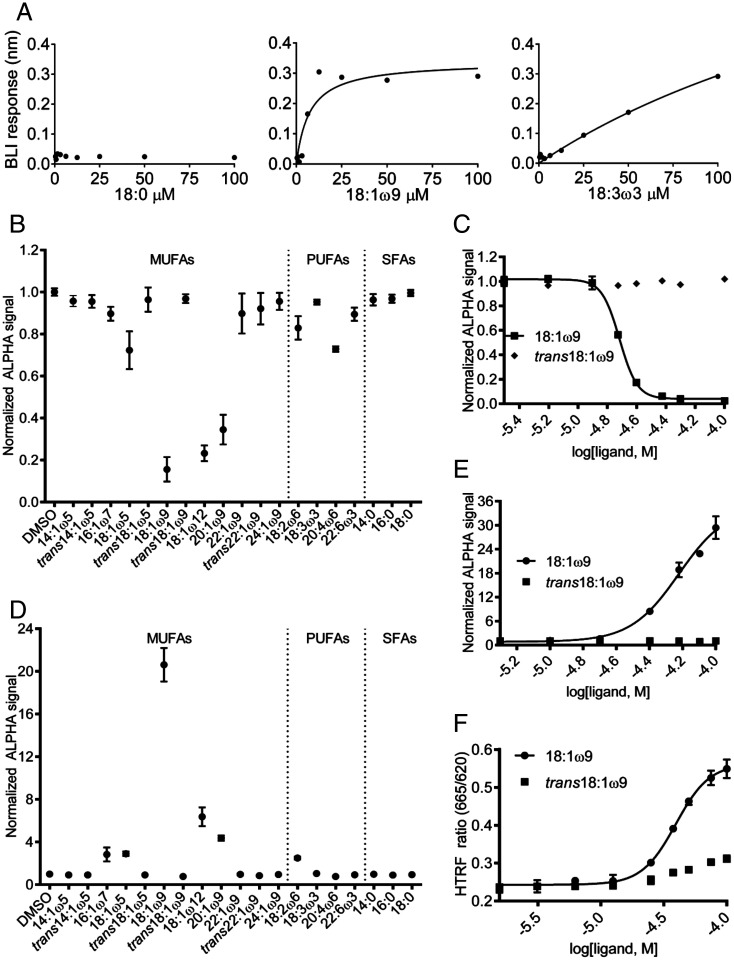
18:1ω9 MUFA binds to TLX ligand-binding domain, reduces the corepressor, and enhances the coactivator interaction. (*A*) Dose-dependent binding response of different classes of fatty acids with the same carbon chain length (18 carbons) to the TLX ligand-binding domain, measured by BLI label-free technology. 18:0 SFA has no observable binding response (*Left*). 18:1ω9 MUFA binding response fit is a saturable one-site fit (the *K_D_* =7.3 µM, 95% CI = 0.385 to 14.2 µM; *Middle*). 18:3ω3 poly-unsaturated fatty acid (PUFA) binding response fit is nonsaturable (*K_D_* = 270.9 µM, 95% CI = 140.8 to 400.9 µM, R*ight*). (*B*) Normalized ALPHA screen for the interaction between TLX ligand-binding domain and a corepressor (atrophin peptide) in the presence of selected MUFAs, PUFAs, and SFAs screened at 25 µM. Signal <1.0 indicates decreased interaction. Note the interaction in the presence of 18:1ω9 versus *trans*18:1ω9. (*C*) Dose-dependent TLX ligand-binding domain/corepressor interaction in the presence of 18:1ω9 (EC_50_ = 19.3 µM, 95% CI = 18.7 to 19.5 µM) or *trans*18:1ω9 (synthetic stereoisomer) MUFA. (*D*) Normalized ALPHA screen of the interaction between TLX ligand-binding domain and a coactivator (NCOA1 peptide) in the presence of selected MUFAs, PUFAs, and SFAs screened at 50 µM. Signal >1.0 indicates increased interaction. Note the interaction in the presence of 18:1ω9 vs. *trans*18:1ω9. (*E*) Dose-dependent TLX ligand-binding domain/coactivator interaction in the presence of 18:1ω9 (EC_50_ = 59.8 µM, 95% CI = 50.6 to 70.8 µM) or *trans*18:1ω9. (*F*) Dose-dependent homogeneous time-resolved fluorescence (HTRF) signal for the TLX/coactivator peptide recruitment in the presence of *cis* vs. *trans* 18:1ω9 MUFA (EC_50_ = 39.3 µM for 18:1ω9, 95% CI = 37.0 to 41.7 µM). See also *SI Appendix*, Figs. S4–S6.

To determine whether fatty acids actually bind to the purified TLX ligand-binding domain (*SI Appendix*, Fig. S5*A*), we used biolayer interferometry (BLI) (32), a biophysical method that has been previously implemented to examine the binding of synthetic small molecules to other nuclear receptors and recently also to the TLX ligand-binding domain (17). We compared the binding responses of different fatty acids with an 18-carbon chain length to the TLX ligand-binding domain (Fig. 2*A*). Stearic acid (18:0 saturated fatty acid) had no observable binding, while α-linolenic acid (18:3ω3 polyunsaturated fatty acid) failed to reach equilibrium or saturate over the concentrations tested, suggesting a nonspecific binding interaction. Oleic acid (18:1ω9 MUFA), however, displayed a saturable binding response with a dissociation constant (*K_D_*) of 7.3 µM, which would not be expected if nonspecific binding interactions were occurring. Interestingly, the synthetic 18:1ω5 MUFA had a similar saturable binding response with a *K_D_* of 6.5 µM (*SI Appendix*, Fig. S5*B*). These experiments show that MUFAs, such as 18:1ω9, can bind to the TLX ligand-binding domain.

### Oleic Acid Binding to TLX Promotes Recruitment of Coactivators.

To understand the functional effects of MUFA binding to TLX, we considered that in its basal nonligand bound state TLX recruits corepressor proteins such as atrophins (ATN 1 and 2) (33), lysine-specific demethylase (LSD1) (34), and histone deacetylases (HDAC 1, 3, and 5) to potently repress transcription (35). We first confirmed the direct interaction between TLX and the atrophin corepressor peptide using BLI and found that the atrophin peptide binds TLX with a *K_D_* of 14.2 µM (*SI Appendix*, Fig. S6*A*). We next used the amplified luminescent proximity homogeneous assay (ALPHA), a proximity assay that measures the interaction of the donor and acceptor beads using luminescence. With atrophin peptide and TLX ligand-binding domain on the donor and acceptor beads, respectively, competition with untagged atrophin peptide attenuated the ALPHA signal with an EC_50_ (effective concentration, 50%) of 1.3 µM, demonstrating that the assay can detect both TLX–corepressor binding and its disruption (*SI Appendix*, Fig. S6*B*). To then determine whether fatty acids could disrupt the TLX–atrophin peptide interaction, we examined a panel of fatty acids (saturated, monounsaturated, and polyunsaturated) (Fig. 2*B*). The saturated fatty acids (14:0, 16:0, and 18:0) had no effect on the ALPHA signal, indicating that they do not interfere with the interaction of atrophin with the TLX ligand-binding domain. The polyunsaturated fatty acids (18:2ω6 and 20:4ω6) were, at best, only modestly active as they minimally diminished the ALPHA signal, indicating that they prevented some interaction of atrophin with the TLX ligand-binding domain. Among the MUFAs, both the short- and long-chain fatty acids (e.g., 14:1ω5 and 24:1ω9) were inactive. The medium-chain MUFAs, however—18:1ω9 (oleic acid), 18:1ω12, and 20:1ω9—clearly disrupted the interaction of TLX with the corepressor. Out of the entire panel of fatty acids examined, *cis* 18:1ω9 (oleic acid) reduced the interaction of atrophin with the TLX ligand-binding domain the most. Given that this was also the most abundant MUFA in human neural stem and progenitor cells (*SI Appendix*, Fig. S1*B*) and in the dentate gyrus in vivo (Fig. 1*B*, *SI Appendix*, Fig. S3), and the most potent fatty acid in the panel at a single dose, we next performed dose–response experiments. These confirmed that 18:1ω9, but not the unnatural *trans* isomer, disrupts the interaction of the TLX ligand-binding domain with the atrophin peptide in a dose-dependent manner, with an EC_50_ = 19.3 µM (Fig. 2*C*). We thus conclude that the TLX ligand-binding domain stereospecifically recognizes certain fatty acids and that occupancy of the TLX ligand-binding pocket by specific MUFAs, especially oleic acid, elicits conformational changes that disengage the atrophin corepressor.

We next wondered whether fatty acid ligands could induce TLX to switch from corepressor to coactivator binding. TLX interacts with nuclear receptor coactivators such as NCOA1, NCOA2, and NCOA3 (SRC1–3) (36). To examine whether oleic acid-bound TLX could recruit NCOA1, we again used the ALPHA screen, placing a tagged NCOA1-II coactivator peptide containing the canonical LXXLL nuclear receptor interaction motif and the TLX ligand-binding domain on donor and acceptor beads, respectively. In the absence of fatty acids or in the presence of only saturated or polyunsaturated fatty acids, there was no interaction between the NCOA1-II peptide and the TLX ligand-binding domain, but 18:1ω9 markedly increased the ALPHA signal (Fig. 2*D*), indicating that the NCOA1-II peptide interacted with the TLX ligand-binding domain. As with disruption of the TLX–corepressor interaction, dose-dependent enhancement of the TLX–coactivator interaction was specific to 18:1ω9 and not *trans*18:1ω9 (Fig. 2*E*), confirming stereospecificity of fatty acid binding and coactivator recruitment, although saturable coactivator peptide binding was not observed under these conditions (Fig. 2*E*). To then determine the affinity of coactivator binding, we performed competition experiments and found that, in the presence of saturating 18:1ω9, TLX recruited both the NCOA1-II peptide and a receptor-interaction domain from NCOA3, with EC_50_ values of 5.6 µM (*SI Appendix*, Fig. S6*C*) and 3.3 µM (*SI Appendix*, Fig. S6*D*), respectively. A TLX-based homogeneous time-resolved fluorescence assay independently verified ligand-dependent coactivator recruitment and the specificity of *cis/trans* 18:1ω9 recognition (Fig. 2*F*). These data strongly indicate that oleic acid binding to TLX ligand-binding domain determines whether TLX functions as a transcriptional repressor or an activator.

**Fig. 3.**
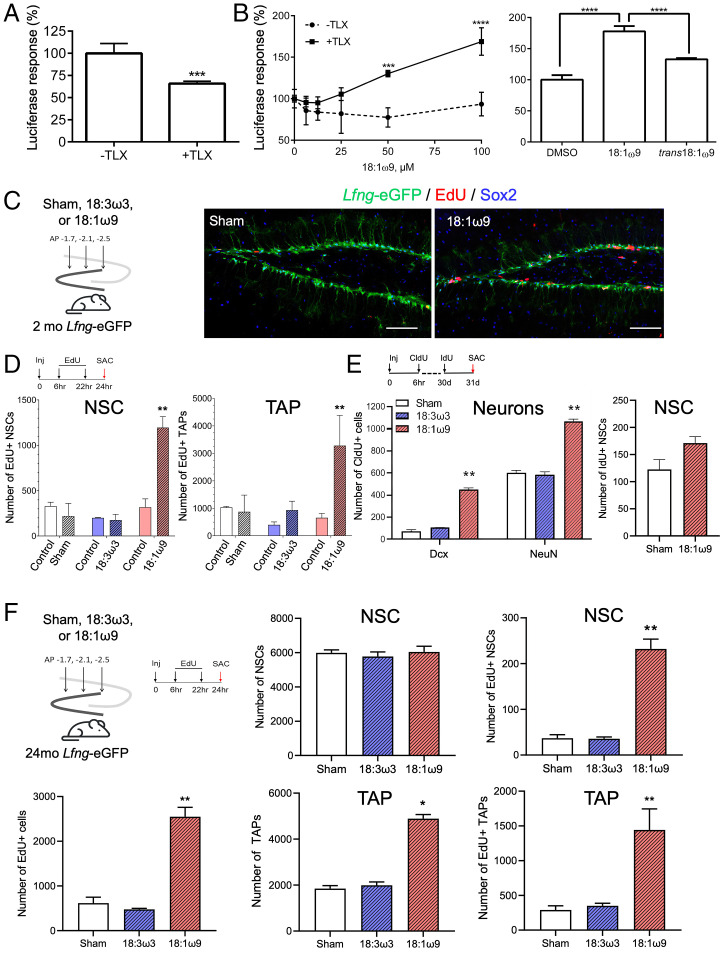
18:1ω9 increases adult neural stem cell proliferation and neurogenesis in vivo in both young and old mice. (*A*) TLX-based dual luciferase reporter activity in HeLa cells without (−TLX) or with (+TLX) TLX-expressing plasmids (*n* ≥ 3). Bar graphs represent mean ± SEM; ***P ≤ 0.005. (*B*) Dose-dependent TLX-luciferase response in HeLa cells with or without TLX-expressing plasmids normalized to the respective vehicle control, DMSO (*Left*). TLX-luciferase response in HeLa cells with TLX-expressing plasmid in the presence of *cis* or *trans* 18:1ω9 (100 µM each) normalized to the respective vehicle control, DMSO (*Right*). Bar graphs represent mean ± SEM. *n* ≥ 3 for all data points. Statistics was done using two-way ANOVA and Tukey's multiple comparisons test to test the difference in normalized signals between HeLa cells with and without TLX. DMSO vs. *trans*18:1ω9 did not significantly differ. ****P ≤ 0.001. (*C*) Two-month-old (mo) *Lfng*-eGFP mice were injected with either sham (empty injection needle), 18:1ω9, or 18:3ω3 fatty acids (300 nL of pure fatty acid per injection site, no solvent) into the left dentate gyrus. Representative confocal photomicrographs show *Lfng*-eGFP^+^ neural stem cells (NSCs), Sox2^+^ transient amplifying neuroprogenitors (TAPs), and EdU^+^ proliferating cells in the subgranular zone of a 2-mo-old *Lfng*-eGFP mice treated with either sham or 18:1ω9. (Scale bar: 100 µm.) (*D*) The absolute number of proliferating NSCs (GFAP+, Sox2+, EdU+) and TAPs (GFAP−, Sox2+, EdU+) in the whole dentate gyrus of 2-mo-old *Lfng*-eGFP mice injected with sham, 18:1ω9, or 18:3ω3 into the left dentate gyrus followed by intraperitoneal injection of EdU (50 mg/kg intraperitoneally, i.p.) 6 h and 22 h following fatty acid injections. Mice were killed (SAC) 2 h after the second EdU injection (*n* = 3 to 5 per group). The right dentate gyrus served as a noninjection control. Sham served as an injection site control. 18:3ω3 served as a fatty acid control. Bar graphs (mean ± SEM) show absolute cell numbers for the respective whole dentate gyrus. For statistical analysis (two-way ANOVA, Tukey’s multiple comparison test), we compared left and right dentate gyrus from the same mouse. (*E*) The absolute number of newborn immature neurons (DCX+ CldU+) and granule cells (NeuN+, CldU+) as well as NSCs (GFAP+, Sox2+, IdU+) in the whole dentate gyrus of 2-mo-old *Lfng*-eGFP mice 30 d after they were injected bilaterally with either sham, 18:1ω9, or 18:3ω3. To trace newborn neurons, CldU (85 mg/kg i.p., 4 injections, 2 h apart) was given 6 h following the fatty acid injection. To trace proliferating NSCs, IdU (85 mg/kg i.p., four injections, 2 h apart) was given 30 d following the fatty acid injection. Mice were killed (SAC) 24 h after the first IdU injection (*n* = 3 to 4 per group). Bar graphs (mean ± SEM) show absolute cell numbers for both whole dentate gyri. Statistics was done using one-way ANOVA and Tukey’s multiple comparison test. (*F*) The absolute number of NSCs (GFAP+, Sox2+), proliferating NSCs (EdU+), TAPs (GFAP-, Sox2+), and proliferating TAPs (EdU+) in the whole dentate gyrus of 24-mo-old *Lfng*-eGFP mice injected bilaterally with either sham, 18:1ω9, or 18:3ω3 fatty acids followed by the intraperitoneal injection of EdU (50 mg/kg i.p.) 6 h and 22 h following fatty acid injection. Mice were killed (SAC) 2 h after the second EdU injection (*n* = 3 to 5 per group). Sham served as an injection site control. 18:3ω3 served as a fatty acid control. Bar graphs (mean ± SEM) show absolute cell numbers for both whole dentate gyri. Statistics was done using one-way ANOVA and Tukey’s multiple comparison test. **P ≤ 0.05. See also *SI Appendix*, Table S1 for quantitative data.

To examine the effect of 18:1ω9 on TLX-mediated transcription, we built a dual luciferase transcriptional reporter system with a previously reported TLX-binding response element (37). We cotransfected a TLX expression vector or empty vector control with the reporter plasmids in HeLa cells grown in media with charcoal-stripped fetal bovine serum. This delipidated serum is expected to minimize exogenous fatty acids and other nonpolar molecules but not endogenous fatty acids produced by lipogenesis. Luciferase expression was modestly reduced in TLX-expressing cells compared to cotransfection with the empty TLX expression vector (Fig. 3*A*), confirming the repressive function of TLX at baseline. We also confirmed the predicted 18:1ω9 agonist activity by the expected dose-dependent increase in luciferase expression (Fig. 3*B*, *Left*). This effect was not seen with *trans*18:1ω9 (Fig. 3*B*, *Right*; *SI Appendix*, Fig. S6*E*). These responses were modest, perhaps due to the presence of endogenous 18:1ω9, but verify that the stereospecific recognition of the *cis* form of 18:1ω9 is retained in living cells and that 18:1ω9 binding promotes TLX-mediated transcriptional activation of TLX-dependent genes.

### Oleic Acid Triggers Neural Stem Cell Mitosis and Neurogenesis In Vivo.

To test the effect of 18:1ω9 on adult neurogenesis in vivo, we took advantage of the *Lfng*-eGFP transgenic mice, in which enhanced green fluorescent protein (eGFP) selectively labels radial neural stem cells (38). We stereotactically delivered either sham, 18:1ω9, or 18:3ω3 (fatty acid control) to the dentate gyrus of young (Fig. 3 *C–E*) or old (Fig. 3*F*) mice, treated them with BrdU analogs to label newborn cells, and killed them at different time points postinjection. Compared to controls, the number of proliferating EdU^+^ neural stem cells and their progeny, transiently amplifying progenitors, in young mice was elevated 24 h postinjection at the site of 18:1ω9 injection only (Fig. 3 *C* and *D* and *SI Appendix*, Table S1*A*). The stimulatory effect of 18:1ω9 is thus specific and local. The number of immature (DCX^+^, CldU^+^) and mature (NeuN^+^, CldU^+^) newborn neurons rose 30 d following 18:1ω9 injection (Fig. 3*E*, *Left* and *SI Appendix*, Table S1*B*), confirming neurogenesis had been stimulated. The number of proliferating (IdU^+^) neural stem cells at this time point did not significantly differ from controls (even though there was an upward trend), indicating that the stimulatory effect of exogenous 18:1ω9 on these cells in young mice is short-lived (Fig. 3*E*, *Right* and *SI Appendix*, Table S1*B*). In aged mice, 18:1ω9 also led to substantial proliferation of neural stem cells and transiently amplifying progenitors (Fig. 3*F* and *SI Appendix*, Table S1*C*). While the total number of GFAP^+^, Sox2^+^ neural stem cells did not change, the number of those dividing (GFAP^+^, Sox2^+^, EdU^+^) significantly increased. This was accompanied by an increased total number of transiently amplifying progenitors (GFAP^−^, Sox2^+^) and those that proliferated (GFAP^−^, Sox2^+^, EdU^+^), further corroborating 18:1ω9 effects observed in the young mice (Fig. 3*D*).

**Fig. 4.**
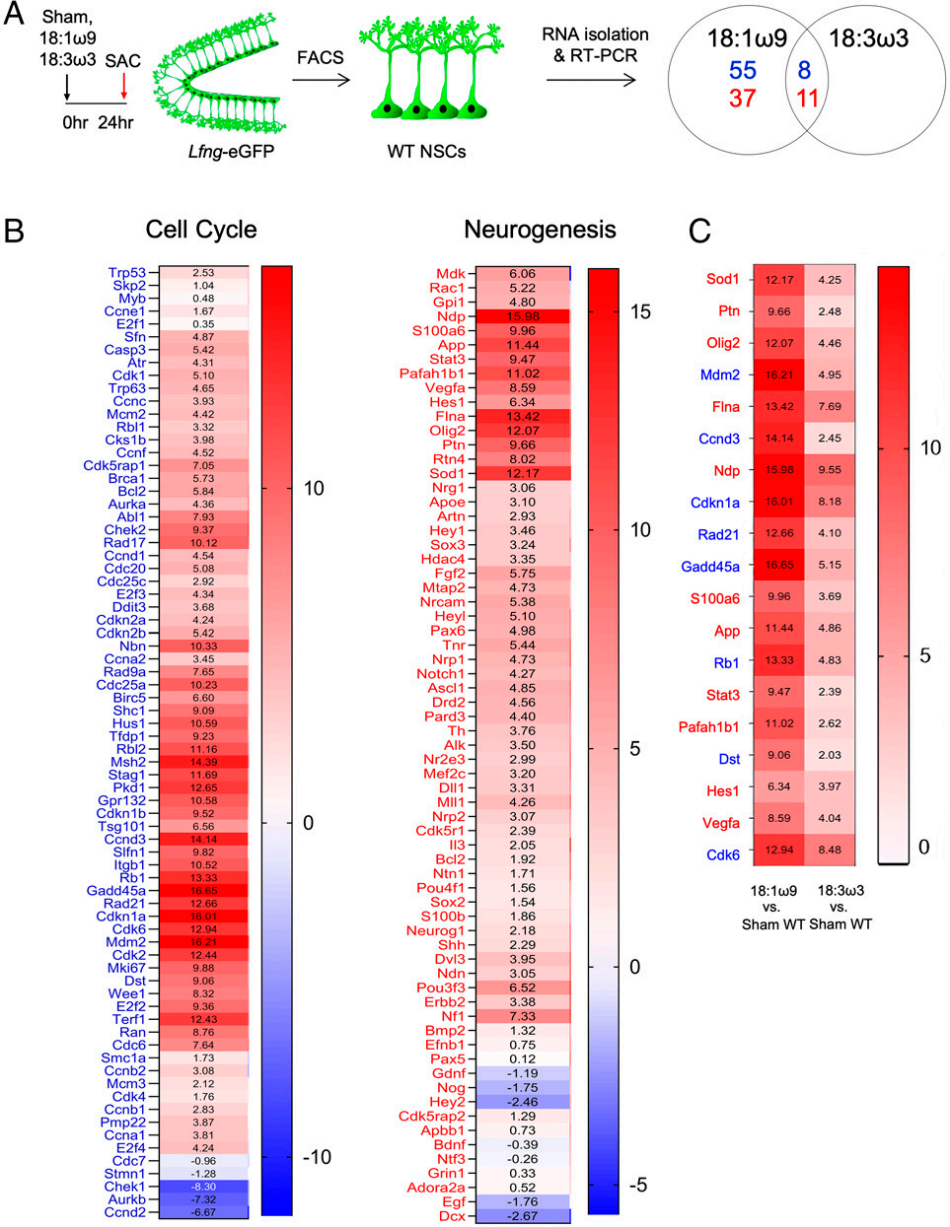
18:1ω9 affects adult murine hippocampal neural stem cell cell cycle and neurogenesis gene expression. (*A*) Two-month-old *Lfng*-eGFP mice—in which all neural stem cells (NSCs) are labeled green—were injected with either sham (empty injection needle), 18:1ω9, or 18:3ω3 (300 nL of pure fatty acid per injection site) (*n* = 3 per group) into both dentate gyri and eGFP^+^ NSCs were sorted 24 h later. RT-PCR was done using a panel of cell cycle and neurogenesis genes. Venn diagram represents differentially regulated genes derived from pairwise ΔC_t_ value comparison across groups. Numbers in blue are cell cycle genes and numbers in red are neurogenesis genes. (*B*) Heat maps of ΔΔCt values (cited in boxes) were derived by pairwise comparisons of 18:1ω9 vs. sham neural stem cells and indicate 74 cell cycle (blue letters) and 67 neurogenesis (red letters) genes detected in all samples. (*C*) Heat map of ΔΔCt values (cited in boxes) indicates 8 cell cycle (blue letters) and 11 neurogenesis (red letters) genes up-regulated by both 18:1ω9 and 18:3ω3. Pairwise comparisons of 18:1ω9 vs. sham wild-type (WT) neural stem cells indicate genes affected by 18:1ω9. Pairwise comparisons of 18:3ω3 vs. sham WT neural stem cells indicate genes affected by 18:3ω3.

We then sought to determine whether 18:1ω9-activated neural stem cell proliferation and neurogenesis correlates with the cell cycle and neurogenic gene expression in vivo. We injected either sham, 18:1ω9, or 18:3ω3 (fatty acid control) into both dentate gyri of *Lfng*-eGFP mice and performed RT-PCR on sorted neural stem cells 24 h later to measure expression of a panel of cell cycle and neurogenesis genes (Fig. 4*A*). In total, we analyzed 74 cell cycle genes and 67 neurogenesis genes (Fig. 4*B*). We then imposed stringent criteria to qualify a gene as being activated in an 18:1ω9-dependent manner: It should be up-regulated fourfold or more by 18:1ω9 and not affected by 18:3ω3. Sixty-three genes involved in the cell cycle and 48 in neurogenesis satisfied the first criterion (Fig. 4*B*). Of those, 8 cell cycle and 11 neurogenesis genes were not only up-regulated by 18:1ω9 but also by 18:3ω3 (which did not affect any of the panel genes on its own) (Fig. 4*C*). We thus excluded these genes from further analyses as those may be nonspecific fatty acid responders.

### Oleic Acid Effects on Neural Stem Cells and Neurogenesis In Vivo are TLX-Dependent.

**Fig. 5.**
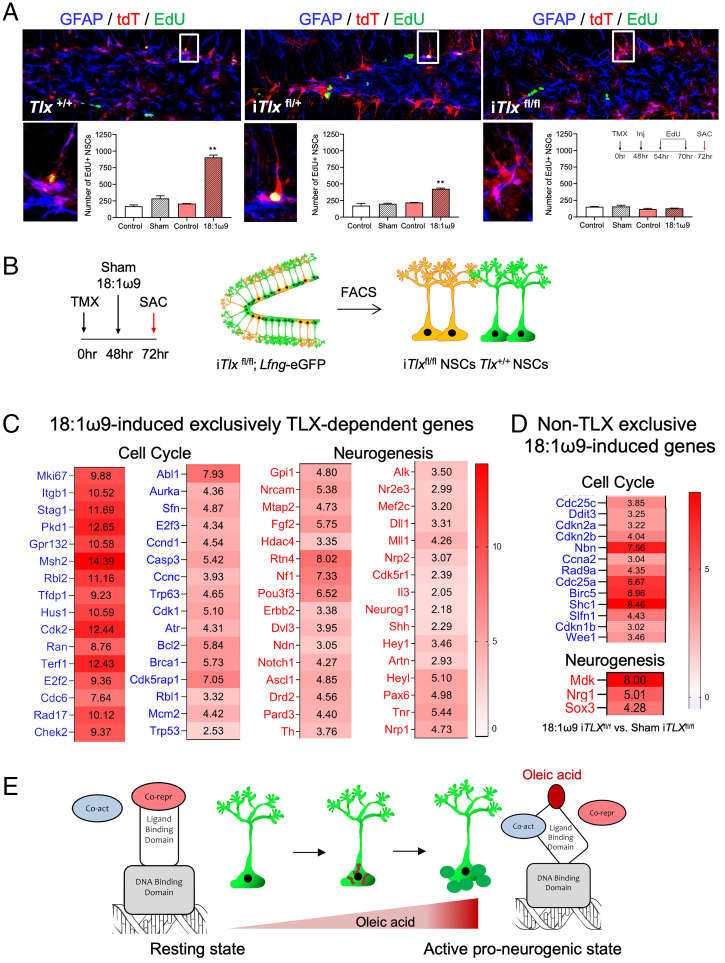
18:1ω9 up-regulates adult murine hippocampal neural stem cell cell cycle and neurogenesis genes in a TLX-dependent manner. (*A*) Two-month-old control *Lfng*-CreER^T2^; RCL-tdT^+/−^ (*Tlx* ^+/+^), *Lfng*-CreER^T2^; RCL-tdT^+/−^; *Tlx* ^fl/+^ (i*Tlx* ^fl/+^), and *Lfng*-CreER^T2^; RCL-tdT^+/−^; *Tlx* ^fl/fl^ (i*Tlx* ^fl/fl^) mice were given tamoxifen (TMX; 120 mg/kg body weight) to induce CreER^T2^ 48 h before injection with either sham (empty injection needle) or 18:1ω9 (300 nL of pure fatty acid per injection site) into the left dentate gyrus, followed by EdU (50 mg/kg i.p.) 6 h and 22 h thereafter. Mice were killed (SAC) 2 h after the second EdU injection (*n* = 3 per group). The right dentate gyrus served as a noninjection control. Sham served as an injection site control. Confocal micrographs show EdU^+^ proliferating cells in mice with two copies of *Tlx* (*Tlx*^+/+^), one copy of *Tlx* (i*Tlx*^fl/+^), and lacking both copies of *Tlx* (i*Tlx*^fl/fl^). TMX-induced cells are labeled with tdT. Boxes are magnified view of a single proliferating TMX-induced NSC (GFAP+, tdT+ EdU+, with clear tdT+ single process emerging from the triangular soma in the subgranular zone) in *Tlx* ^+/+^ and i*Tlx* ^fl/+^ mice. In i*Tlx* ^fl/fl^ mice, magnified region shows nonproliferating TMX-induced NSCs. Proliferating noninduced cells are labeled green (EdU). Bar graphs (mean ± SEM) show the absolute number of proliferating NSCs (GFAP+, EdU+, with clear single process emerging from the triangular soma in the subgranular zone) in the whole dentate gyrus of *Tlx*^+/+^, i*Tlx*^fl/+^, and i*Tlx*^fl/fl^ mice. For statistical analysis (two-way ANOVA, Tukey’s multiple comparison test), we compared left and right dentate gyrus from the same mouse. **P ≤ 0.001. See *SI Appendix* and *SI Appendix*, Fig. S7 for generation of i*Tlx*^fl/+^and i*Tlx*^fl/fl^ mice. (*B*) Two-month-old i*Tlx*^fl/fl^; *Lfng*-eGFP (*Lfng*-CreER^T2^/RCL-tdT/*Lfng*-eGFP/*Tlx*^fl/fl^) mice were given tamoxifen (TMX; 120 mg/kg body weight) 48 h before either sham or 18:1ω9 were injected into both dentate gyri (*n* = 3 per group). i*Tlx*^fl/fl^ NSCs (tdT^+^ eGFP^+^; orange) and *Tlx*^+/+^ NSCs (eGFP^+^) were sorted 24 h following sham/18:1ω9 injection. RT-PCR was done for the same cell cycle and neurogenesis genes as in Fig. 4. (*C*) Heat maps of ΔΔC_t_ values (cited in boxes) show 32 cell cycle and 32 neurogenesis genes exclusively up-regulated by 18:1ω9 (fourfold or greater change) in a TLX-dependent manner. These genes were 1) activated by 18:1ω9 (pairwise comparison of 18:1ω9-treated vs. sham-treated *Tlx*^+/+^ NSCs (*Lfng*-eGFP^+^ NSCs from *Lfng*-eGFP mice; Fig. 4*B*), 2) derepressed in i*Tlx*^fl/fl^ NSCs (pairwise comparison of sham i*Tlx*^fl/fl^ NSCs (eGFP^+^; tdT^+^) vs. sham *Tlx*^+/+^ NSCs (eGFP^+^); see *SI Appendix*, Fig. S8), and 3) not up-regulated in the absence of TLX (pairwise comparison of 18:1ω9-treated i*Tlx*^fl/fl^ NSCs (eGFP^+^; tdT^+^) vs. sham i*Tlx*^fl/fl^ NSCs (eGFP^+^; tdT^+^); see *D* and *SI Appendix*, Fig. S9). (*D*) Heat maps of ΔΔC_t_ values (cited in boxes) show 13 cell cycle and 3 neurogenesis genes that were up-regulated by 18:1ω9 in the absence of *Tlx* (pairwise comparison of 18:1ω9-treated vs. sham-treated i*Tlx*^fl/fl^ eGFP^+^ tdT^+^ NSCs; see also *SI Appendix*, Fig. S9). (*E*) Proposed model of the 18:1ω9, oleic acid, action as a critical signaling metabolite in NSCs that activates their cell cycle and neurogenesis upon binding to TLX. See also *SI Appendix*, Table S1 for quantitative data.

To determine whether 18:1ω9 requires TLX to affect neural stem cell function, we used *Lfng-*CreER^T2^;RCL-tdT mice (38) and crossed them with the *Tlx*^loxp/loxp^ mice (13) to achieve homozygous deletion of *Tlx* in Cre-induced neural stem cells following tamoxifen administration (*SI Appendix*, Fig. S7). In the resulting i*Tlx*^fl/fl^ mice, wild-type neural stem cells (no Cre activation) have two copies of *Tlx* (*Tlx*^+/+^) while mutant neural stem cells (Cre-induced) lack both copies of *Tlx* (*Tlx*^fl/fl^) and are labeled red (tdTomato, tdT). Thus, in i*Tlx*^fl/fl^ brains we can examine 18:1ω9-dependent cell-autonomous effects in both wild-type and mutant neural stem cells from the same dentate gyrus. The total number of proliferating EdU^+^ neural stem cells in sham-treated i*Tlx*^fl/fl^ mice was lower compared to *Tlx*^+/+^ mice, confirming the expected phenotype of i*Tlx*^fl/fl^ mice (Fig. 5*A* and *SI Appendix*, Table S1*D*). Following 18:1ω9 injection into the dentate gyrus (the same stereotactic coordinates as in Figs. 3 and 4*A*), the number of proliferating EdU^+^ neural stem cells correlated with *Tlx* gene dosage: It was much higher when two copies of *Tlx* were present (*Tlx*^+/+^) than when only one copy was present (i*Tlx*^fl/+^), and 18:1ω9 exerted no proliferative effect when both copies of *Tlx* were absent (i*Tlx*^fl/fl^; Fig. 5*A* and *SI Appendix*, Table S1*D*). Thus, independent of any purely metabolic role for MUFA, the stimulatory effects of exogenous 18:1ω9 on adult hippocampal neural stem cells and neurogenesis are mediated through TLX.

To then determine which of the 18:1ω9-dependent genes are also TLX-dependent, we crossed the i*Tlx*^fl/fl^ mice with the *Lfng*-eGFP mice (i*Tlx*^fl/fl^; *Lfng*-eGFP), in which wild-type *Tlx*^+/+^ neural stem cells are labeled green (eGFP) while mutant *Tlx*^fl/fl^ neural stem cells are labeled both red (tdT) and green (eGFP). This mouse model thus allows us to sort both wild-type and mutant neural stem cells from the same dentate gyrus (Fig. 5*B*). We injected either sham or 18:1ω9 into both dentate gyri 48 h after tamoxifen treatment of i*Tlx*^fl/fl^ mice and performed RT-PCR on sorted neural stem cells 24 h later using the same gene panels as before (Fig. 5*B*). Compared to wild-type neural stem cells (*Tlx*^+/+^ eGFP^+^, tdT^−^) from sham-treated i*Tlx*^fl/fl^; *Lfng*-eGFP mice, neural stem cells lacking *Tlx* (*Tlx*^fl/fl^ eGFP^+^, tdT^+^) had 56 cell cycle and 63 neurogenesis genes up-regulated fourfold or more (*SI Appendix*, Fig. S8). These genes are thus considered to be suppressed by TLX. Among these, 45 cell cycle and 35 neurogenesis genes overlapped with those up-regulated by 18:1ω9 (Fig. 4*B*). Of those, however, 13 cell cycle and 3 neurogenesis genes were up-regulated by 18:1ω9 in the absence of *Tlx* (18:1ω9 vs. sham-treated *Tlx*^fl/fl^ eGFP^+^ tdT^+^ neural stem cells; Fig. 5*D* and *SI Appendix*, Fig. S9), and we excluded them from further analyses as these may not be exclusively TLX-dependent. The remaining 32 cell cycle and 32 neurogenesis genes were up-regulated by 18:1ω9 in wild-type mice (Fig. 4*B*) but not in the absence of TLX (Fig. 5*D*), rendering them exclusively 18:1ω9-activated TLX-dependent genes (Fig. 5*C*).

The 18:1ω9-activated TLX-dependent genes included previously reported TLX targets such as *Ascl1* (13), *Trp53* (39), *Mcm2*, and *Ccnd2* (40) but also revealed new targets such as *Olig2*, *Shh*, *Fgf2*, *Dvl3*, *Hey1*, *Pax6*, *Nf1*, *Dll1*, *Nr2e3*, *E2f2*, *E2f3*, *Cdk1*, and *Cdk2*. It is worth noting that TLX does not simply act as a binary on–off switch: 18:1ω9-bound TLX activates cell cycle genes more strongly than neurogenesis genes, while lack of TLX derepresses neurogenesis genes more strongly (*SI Appendix*, Fig. S8). This is consistent with previous reports that a gradual increase—and not a steady, high expression—of neurogenic genes, such as *Ascl1*, is necessary for neuroprogenitors to slowly enter the cell cycle to avoid premature differentiation (41–43).

## Discussion

In this study we identified oleic acid as a previously unreported endogenous agonist ligand of TLX, whose binding causes TLX to release its corepressor, recruit a coactivator, activate transcription, and through the action of downstream genes increase neural stem and progenitor proliferation and neurogenesis in both young and old mice. These results support a model in which 18:1ω9, oleic acid, acts as a critical signaling metabolite in neural stem cells (Fig. 5*E*). In its ligand-free state, TLX favors corepressor binding and repression of its target genes, causing neural stem cells to remain quiescent. Upon activation of fatty acid synthesis, oleic acid starts to be synthesized and accumulates until it reaches a threshold required as a metabolic prerequisite for the cell cycle entry. At that time, oleic acid binds to TLX, disengaging corepressors and recruiting transcriptional coactivators, which then triggers neural stem cell proliferation. In response to acutely increased oleic acid levels, robust up-regulation of cell cycle genes would prompt neural stem cells to generate progeny while preventing their direct differentiation. Temporally cued derepression of neurogenesis genes could then ensure that the progeny properly proceed into the neurogenic and not astrocytic lineage. We thus propose that neural stem cells use TLX to control the switch between repression and activation in response to neurogenic signals, at the same time tightly balancing the expression of functionally distinct cell cycle and neurogenesis genes. Because TLX activation requires relatively high levels of oleic acid, neural stem cells are not perpetually dividing and quickly depleting in number. Therefore, oleic acid might not only be essential for neural stem cell proliferation and neurogenesis in the hippocampus but also for the preservation of neural stem cell population over time. Testing aspects of this model will be of intense interest to many fields of biology.

Our findings shed light on the importance of fatty acid metabolism and MUFAs as signaling molecules in neural stem and progenitor cells. As previously reported, fatty acid oxidation is high in quiescent stem cells but down-regulated in proliferating cells. Several reports suggest that adult neural stem cells shift their metabolism to activate de novo lipogenesis in order to proliferate. For example, in one study Knobloch et al. reported that quiescent neural stem cells depend on fatty acid oxidation as they specifically express carnitine palmitoyl transferase (Cpt1aA) (44), a key enzyme required for the transfer of carnitine to mitochondria and thus fatty acid oxidation. In another study, Knobloch et al. reported that neural stem cells require fatty acid synthase-dependent buildup of lipids in order to proliferate (21). Further, malonyl-coenzyme A plays an important role in this process as a rate-limiting substrate for the de novo fatty acid synthesis and an inhibitor of fatty acid oxidation via Cpt1a (45). Interestingly, hematopoetic stem/progenitor cells (46) and adult hippocampal neural stem cells (44) treated with malonyl-Coenzyme A proliferate more than control, suggesting the critical importance of this metabolic shift from fatty acid oxidation to de novo fatty acid synthesis for stem cell proliferation. Thus, detection of the increased intracellular levels of de novo fatty acid synthesis products such as C16 palmitate and other middle- and high-chain fatty acids, such as oleic acid, by the cell and specifically TLX to trigger the cell cycle may play a critical regulatory role in the checkpoint mechanisms that allow further progression of the cell cycle program.

In sum, we report an endogenous ligand of TLX whose production is regulated within the neural stem cells where TLX resides. Other exogenous natural and synthetic TLX ligands that must be obtained through the diet (such as retinoid from vitamin A) have been reported (16), but their impact on neurogenesis or other physiologic processes in vivo remains to be determined. The observation that exogenous oleic acid treatment is sufficient to stimulate TLX-dependent neurogenesis in vivo lays a foundation for future efforts to preserve neural stem cells during aging and in disease. Impaired hippocampal neurogenesis has been implicated in a variety of neurological and psychiatric diseases, and the ability to stimulate neurogenesis directly could lead to novel and better-tolerated treatments for these diseases. The discovery of a functional endogenous TLX ligand motivates efforts to identify either brain-penetrant small molecules that can modulate TLX function or strategies to increase endogenous oleic acid and promote cell-based repair and neural regeneration.

## Materials and Methods

A full description of material and methods is available in *SI Appendix*.

### Reagents, Cell Models, and Mouse Models.

Fatty acids were purchased from commercial sources and used without further purification except for 18:1ω5 and *trans*18:1ω5, which were synthesized in-house. Human neural stem and progenitor cells were generated from WA09 (H9) human embryonic stem cells maintained on Matrigel-coated plates in Essential 8 Medium (47), using a variation of the dual SMAD inhibition protocol (48–50). HeLa cells were obtained from the tissue culture core at Baylor College of Medicine. *Lfng*-eGFP mice (RRID:MMRRC_015881-UCD) were obtained from GENSAT (51) and received as FVB/N-C57BL/6 hybrids. They were crossed to C57BL/6J mice for at least 10 generations and fully characterized prior to use (38). *Tlx*^loxp/loxp^ mice were a gift from Ronald Evans, Salk Institute for Biological Sciences, San Diego, CA (13). C57BL/6J mice and the AI14 (RCL-tdT) reporter line were from The Jackson Laboratory (JAX 000664; RRID:IMSR_ JAX:000664 and JAX 007908; RRID:IMSR_JAX:007908, respectively) (52). *Lfng*-CreER^T2^ mice were generated in the M.M.-S. laboratory (38). All mouse experiments were done according to the Baylor College of Medicine Institutional Animal Care and Use Committee approved protocol (AN-5004).

### Chemistry and Binding Analyses.

Proton NMR (^1^H-NMR), gas chromatography–mass spectrometry, and imaging mass spectrometry were done according to established protocols (53, 54). We used BLI, an optical label-free technology, to examine interactions between the TLX LBD and different fatty acids. BLI binding measurements were performed with 6xHis-TLX LBD using the Octet Red 96 instrument (Ni-NTA Biosensors; catalog no. 18-5103, FortéBio Inc./Molecular Devices LLC). Alpha screen assays were performed following the manufacturer’s protocol (AlphaScreen Histidine (Nickel Chelate) and AlphaScreen GST Detection Kit, catalog nos. 6760619C and 6760603C; PerkinElmer) with some modifications. The homogenous time-resolved fluorescence assay was carried out in low-volume 384-well plates at room temperature using Anti-His-Terbium (Tb) antibody and streptavidin-d2 (CisBio/PerkinElmer).

### Mouse Model Experiments.

To induce Cre, tamoxifen (120 mg in 10 mL of 1:9 ethanol:corn oil mixture) solution was administered intraperitoneally to *Lfng*-CreER^T2^-based mice. Control mice were injected with ethanol:corn oil mixture only. Stereotactic injections were done to anesthetized mice as per approved protocol (AN-5004). We delivered fatty acids into the right dentate gyrus at three sites relative to Bregma: anteroposterior (AP) −1.5 mm, −1.7 mm, or −1.9 mm; lateral–lateral (LL) −1.6 mm; and dorsoventral (DV) −1.9 mm, using nanoinjector (Nanoject II; Drummond Scientific). Each site received 305.4 nL of pure fatty acids in six pulses (50.9 nL per pulse). Injection was done in a slow delivery mode over 2 s per pulse. Each pulse was separated by 15 s. A glass capillary was gradually moved in and retracted from the tissue, over 3 min. Following killing at a given time point, mice were either immunostained according to the published protocol (38) or dentate gyri were isolated (55) and cells sorted using BD FACS Aria II. Each sample was generated from two dentate gyri. RNA was isolated from sorted cells using RNeasyplus Micro kit (Qiagen). RT-PCR was performed using RT2 profiler PCR array Mouse Neurogenesis (catalog no. PAMM-404Z; Qiagen), Mouse Cell Cycle (catalog no. PAMM-020Z; Qiagen), Mouse Notch Signaling Pathway (catalog no. PAMM-059Z; Qiagen), and RT2 SYBR Green qPCR master mix (catalog no. 330513; Qiagen). Expressions were normalized to β-2 macroglobulin (B2m) gene. We calculated fold changes compared to control group using the ΔΔCt method.

### Statistical Analysis.

Statistical analysis was performed using GraphPad Prism 8.0 (GraphPad RRID:SCR_002798). The sample size was determined based on published data (56, 57). Experiments involving two groups were compared using unpaired Student *t* test. Experiments involving more than two groups with one variable were compared by one-way ANOVA or two-way ANOVA followed by Tukey’s honestly significant difference post hoc test analysis for pairwise comparisons. Significance was defined as *P* < 0.05.

## Supplementary Material

Supplementary File

## Data Availability

All study data are included in the article and/or *SI Appendix*.
